# Supplementary material for: Effects of fluid shear stress duration on the mechanical properties of HeLa cells using atomic force microscopy
Source: PLoS One. 2025 May 5;20(5):e0321296. doi: 10.1371/journal.pone.0321296 (PMC12052195; doi:10.1371/journal.pone.0321296)
Supplement: Attachment — Submitted filename: Reviewer Report.pdf [file pone.0321296.s001.pdf]

Reviewer 1: This manuscript investigates the impact of fluid shear stress (FSS) duration on the mechanical properties of HeLa cells, presenting valuable insights into the field. However, several aspects require further clarification and improvement to enhance the manuscript's scientific rigor and comprehensiveness.
1. For introduction, the authors should systematically summarize the key findings from previous research on the effects of FSS on cells, especially those relevant to cancer cells. Additionally, how do the current study's objectives build upon or address the limitations of these prior works? A more coherent connection would strengthen the introduction.

2. Were there any quality control measures in place to ensure the consistency and stability of the HeLa cell line throughout the experiments? The authors should explain why DMEM-H medium was specifically chosen over other commonly used media for culturing HeLa cells?

3. Although the dimensions and assembly of the parallel - plate flow chamber are described, the selection of these specific dimensions lacks justification. How were these dimensions determined? Were there any preliminary simulations or experiments to optimize the chamber design for the intended fluid shear stress applications? Additionally, it would be helpful to know more about the material of the flow chamber and its potential impact on cell adhesion and the fluid flow characteristics.

4. the authors could provide more details on the validation of the fluid shear stress calculation method. Were there any experimental measurements to corroborate the calculated values?
5. Were multiple cells measured for each experimental condition, and if so, what was the level of variation in the measured mechanical properties? Additionally, the choice of the Sneddon model for analyzing the force - displacement curves should be justified. Are there any limitations of this model when applied to HeLa cells, and were other models considered?

6. The manuscript describes the changes in cell morphology and mechanical properties separately. It would be interesting to explore if there is a direct correlation between the two. could the authors perform a correlation analysis between the cell height and the Young's modulus to determine if changes in one property are associated with changes in the other?
7. The authors discuss potential mechanisms underlying the observed changes in cell mechanics, such as cytoskeletal reorganization, alterations in cell membrane fluidity, and changes in intracellular signal transduction. However, these discussions are rather speculative. Are there any experimental data or references to support these proposed mechanisms?

8. the connection between the mechanical properties measured in vitro and the actual metastatic behavior of HeLa cells in vivo remains unclear. Could the authors discuss how these in vitro findings might translate to the in vivo context? Are there any in vivo studies that support the relationship between mechanical properties and metastasis?

9. Recent work in the field of Mechanical Properties of Cells should be cited in introduction, such as: https://doi.org/10.1016/j.biotechadv.2020.107648; DOI: 10.1017/S1431927622000290; DOI: 10.1039/D0LC00477D; doi: 10.3791/50497

Reviewer 2: The mansucript presents an experimental study on how cells change their mechanics after being exposed to hydrodynamic flow. This is an interesting and relevant subject, and the results are novel. That said, the study does suffer from some major issues that need to be improved, before publication can be recommendend. These are, in order of relevance:
1) It is not clearly said, but the way I understand the manuscript, each experiment is carried out for a single cell only. The error bars appear to stem from different measurement points on the same cell. Measuring AFM at different points on the same cell is highly important and a good point of the paper. However, we can expect significant cell-to-cell variation as well. Therefore, it is highly important to make

statistics on, at least, 5-10 different cells for each experimental situation.
2) I am missing a quantitative connection to earlier works. The HeLa cells used in the manuscript are a widely-used cell line and I am sure there exist earlier AFM (and possibly other) measurements of their elastic properties in the absence of flow. The authors should look for such data and compare their control experiment without flow to these works.
3) The shear stress calculation in 2.3 1) seems to be an approximation. The exact flow field for a rectangular geometry is well-known, but is a somewhat lengthy expression containing an infinite convergent sum as given in standard texts on hydrodynamics. Please state which approximations were made to arrive at the simple form given in 2.3 1).
