# Supplementary material for: Effects of fluid shear stress duration on the mechanical properties of HeLa cells using atomic force microscopy
Source: PLoS One. 2025 May 5;20(5):e0321296. doi: 10.1371/journal.pone.0321296 (PMC12052195; doi:10.1371/journal.pone.0321296)
Supplement: Attachment — Submitted filename: Response to Reviewers.docx [file pone.0321296.s002.docx]

**Response to Reviewers**

Dear editor and reviewers,

Thank you for offering us an opportunity to improve the quality of our submitted manuscript (Effects of Fluid Shear Stress Duration on the Mechanical Properties of HeLa Cells using Atomic Force Microscopy). We appreciated very much the reviewers’ constructive and insightful comments. In this revision, we have addressed all of these comments/suggestions. We hope the revised manuscript has now met the publication standard of your journal.

We highlighted all the revisions in red colour.

On the next pages, our point-to-point responses to the queries raised by the reviewers are listed.

**Reviewer 1:** This manuscript investigates the impact of fluid shear stress (FSS) duration on the mechanical properties of HeLa cells, presenting valuable insights into the field. However, several aspects require further clarification and improvement to enhance the manuscript's scientific rigor and comprehensiveness.

Comment 1. For introduction, the authors should systematically summarize the key findings from previous research on the effects of FSS on cells, especially those relevant to cancer cells. Additionally, how do the current study's objectives build upon or address the limitations of these prior works? A more coherent connection would strengthen the introduction.

Response: Thanks for the suggestions. We have revised this part in the manuscript to enhance its clarity and depth. Below is our response to the issues you raised and the corresponding modifications. Page 3, line 58 to 110. In the revised manuscript, we have expanded the introduction section to systematically summarize previous studies on the effects of FSS on cancer cells and clarified how the current research advances further based on these works. Through these revisions, we have enhanced the coherence and scientific rigor of the introduction, highlighting the novelty and significance of the current study in building upon prior research.

Comment 2. Were there any quality control measures in place to ensure the consistency and stability of the HeLa cell line throughout the experiments? The authors should explain why DMEM-H medium was specifically chosen over other commonly used media for culturing HeLa cells?

Response: Thank you for your question regarding the quality control measures for the HeLa cell line. The relevant modifications have been made in the manuscript on page 19, lines 397-402.

To ensure the consistency and stability of HeLa cells, all cells were obtained from the Cell Bank of the Chinese Academy of Sciences, ensuring a uniform genetic background and absence of contamination. To eliminate the impact of cell passage batch variations on the measurement results, all experiments were conducted using cells from the same passage. The cells were cultured under identical conditions, and the application of fluid shear stress in the in vitro system was performed in the same environment. Additionally, five independent experiments were carried out to ensure the reliability of the results. We strictly controlled the culture environment, monitored cell morphology, and excluded abnormal differentiation or apoptosis. All procedures, including cell culture and shear stress experiments, were carried out in a 37°C, 5% CO₂ incubator, with standardized passage density, trypsin digestion time, and medium replacement frequency.

In the revised manuscript, we have explained why DMEM-H medium was specifically chosen over other commonly used media for culturing HeLa cells (page 7, lines 137 to 140). DMEM-H was chosen as the culture medium because it is the standard for HeLa cell culture. Its high glucose concentration (4.5 g/L) meets the high metabolic demands of HeLa cells, better supports their growth and proliferation, prevents stress responses caused by nutrient deficiency, and maintains energy metabolism balance under shear stress conditions.

Comment 3. Although the dimensions and assembly of the parallel - plate flow chamber are described, the selection of these specific dimensions lacks justification. How were these dimensions determined? Were there any preliminary simulations or experiments to optimize the chamber design for the intended fluid shear stress applications? Additionally, it would be helpful to know more about the material of the flow chamber and its potential impact on cell adhesion and the fluid flow characteristics.

Response: Thank you for your attention to the design details of the parallel-plate flow chamber. Here is our detailed response to the questions you raised:

The parallel-plate flow chamber is a commonly used model for studying cellular mechanical properties in vitro and is widely employed to simulate the physiological environment of cells under dynamic fluid shear stress (FSS). In this study, we referred to the mature product design from GlycoTech, as shown in Figure 1, and selected a rectangular flow chamber with a width of 1 cm and a length of 4 cm. The main reason for choosing this size is that the larger experimental area (1 cm × 4 cm) can provide a greater number of cell samples, which facilitates the extraction of sufficient cells for subsequent experiments (such as gene expression and protein analysis) after shear stress application. This avoids the risk of experimental interruptions due to insufficient sample quantities in circular flow chambers.

**Fig. 1: Parallel-Plate Flow Chamber from GlycoTech**

To ensure the rationality of the flow chamber design and the accuracy of the experiments, we conducted computational fluid dynamics (CFD) simulations. The simulation results show that within the target shear stress range (e.g., 0.1-10 dyn/cm²), the flow velocity distribution in the flow chamber is uniform, with fully developed boundary layers and shear stress errors of less than 1%, meeting the experimental accuracy requirements. Combining the CFD results with the classical parallel-plate laminar flow theoretical formula further confirms the rationality of the dimension parameters.

The main body of the flow chamber is made of polycarbonate, which has the advantages of good biocompatibility, high optical transparency (convenient for microscopic observation), and strong chemical stability (tolerant to cell culture media and sterilization processes).

Although the material of the flow chamber may have potential effects on cell adhesion and fluid flow properties, all experimental groups in this study used flow chambers made of the same material, eliminating the interference of material differences on cell adhesion and fluid properties. This ensures a clear causal relationship between shear stress exposure time (the only variable) and cellular response.

By referring to mature designs, validating with CFD simulations, and strictly controlling material consistency, we ensured the functionality and experimental reliability of the parallel-plate flow chamber. The material properties of the parallel-plate flow chamber may affect initial cell adhesion and flow field boundary conditions, which provides an important direction for our future research. We will further explore the mechanisms by which material properties affect the cellular mechanical microenvironment.

Comment 4. the authors could provide more details on the validation of the fluid shear stress calculation method. Were there any experimental measurements to corroborate the calculated values?

Response: We thank you for your valuable feedback regarding the validation of the fluid shear stress calculation method. We fully agree that providing detailed validation is crucial to ensuring the reliability of the research results. Below is our response to the issues you raised and the corresponding modifications. Page 10, line 196 to 201. Page 16, line 338to 349.

To validate the fluid shear stress calculation method, we combined theoretical formulas with numerical calculations. We used the classical fluid shear stress theoretical formula for computation and compared the results with numerical calculations. The formula is as follows:

1) Fluid Shear Stress Calculation Formula:

$$\tau_{w}=6\mu Q/a^{2}b$$

$\tau_{w}$: Shear stress, in dynes per square centimeter (dyn/cm^2^);

$\mu$：Fluid viscosity, in poise (P);

a: Channel height, in centimeters (cm);

b: Channel width, in centimeters (cm);

Q: Liquid flow rate, in milliliters per second (ml/s).

In this study, the channel height a=0.3 cm,*a*, the channel width  *b*=1cm, and the fluid viscosity  *μ*=1P. Based on the theoretical formula, we calculated the flow rates *Q* corresponding to four different shear stress levels: 5, 10, 15, and 20 dyn/cm². The corresponding flow rates were 0.075, 0.15, 0.225, and 0.3 ml/s, respectively.

For each flow rate parameter, we computed the theoretical shear stress values and compared them with the numerical calculation results. The specific results are shown in the Table 1 below:

**Table 1. Comparison of Theoretical and Numerical Fluid Shear Stress Results**

| **Flow Rate Q (ml/s)** | **Theoretical Shear Stress**  **τw (dyn/cm²)** | **Numerical Shear Stress**  **τw (dyn/cm²)** | **Error** |
| --- | --- | --- | --- |
| 0.075 | 5 | 5.01 | ＜1% |
| 0.15 | 10 | 10.04 | ＜1% |
| 0.225 | 15 | 15.05 | ＜1% |
| 0.3 | 20 | 20.05 | ＜1% |

As shown in Table 1, the error between the theoretical and numerical results is less than 1% in all cases. This demonstrates that our fluid shear stress calculation method is highly accurate and can be reliably applied in fluid shear stress experiments.

Comment 5. Were multiple cells measured for each experimental condition, and if so, what was the level of variation in the measured mechanical properties? Additionally, the choice of the Sneddon model for analyzing the force - displacement curves should be justified. Are there any limitations of this model when applied to HeLa cells, and were other models considered?

Response: We thank the reviewer for their question. During the experiments, we actually measured multiple cells under each experimental condition. However, this was not explicitly stated in the experimental methods section of the original manuscript. To obtain statistically significant results, we performed morphological imaging on one group of cells and measured force curves for five different groups of cells under each experimental condition (the experimental methods section has been updated in the manuscript. Page 19, line 397 to 402，Page19，line406 to 411). The Young's modulus measurement results for multiple groups of cells, obtained using AFM, are shown in Fig. 2

**Fig. 2 Measurement of Young's modulus for multiple groups of cells using AFM**

We measured force curves for five groups of cells under each experimental condition and fitted 20 force curves from each group to determine the Young’s modulus of the cells. （Page22,line457-459.）The experimental results indicate that under identical fluid shear stress conditions, the Young’s modulus values of the cells tend to decrease with increased duration of exposure. As shown in Fig.3.

**Fig. 3 Measuring the Young's modulus of Hela cells subjected to different durations of fluid shear stress by Atomic Force Microscopy (AFM)**

In the revised manuscript, we have explained the rationale for choosing the Sneddon model to analyze the force-displacement curves, and discussed the potential limitations of this model when applied to HeLa cells, as well as whether other models were considered. The detailed explanation is located on Page 13, lines 263-278.

The force curve is converted into an indentation curve based on the contact point on the force curve. Subsequently, the indentation curve is fitted using theoretical models to obtain the Young's modulus of the cells. Currently, the main models available for extracting the Young's modulus of cells are the Hertz-Sneddon, JKR (Johnson-Kendall-Roberts), and DMT (Derjaguin-Muller-Toporov) models. The Hertz-Sneddon model neglects the forces in the contact area between the tip and the cell, such as electrostatic forces, adhesive forces, and frictional forces. In contrast, the JKR and DMT models consider the adhesive forces within the contact area. The JKR model is suitable for situations where the tip is large and the adhesive force between the tip and the sample is significant, while the DMT model is applicable when the tip is small and the adhesive force is minimal. However, the most widely used model in practice is the Hertz-Sneddon model. The Hertz-Sneddon model is based on a series of assumptions about the sample being probed, such as isotropy, homogeneity, linear elasticity, axisymmetry, infinite thickness, and smooth surface. Although cells do not strictly meet these conditions, studies have shown that the Hertz model is applicable when the indentation depth is less than 10% of the sample thickness. Therefore, this study employs the Sneddon model to fit the indentation curves, thereby obtaining the cellular Young's modulus.

Comment 6. The manuscript describes the changes in cell morphology and mechanical properties separately. It would be interesting to explore if there is a direct correlation between the two. could the authors perform a correlation analysis between the cell height and the Young's modulus to determine if changes in one property are associated with changes in the other?

Response: We thank the reviewer for their attention to the correlation between cell morphology and mechanical properties. We agree on the importance of exploring the relationship between cell height and Young's modulus and have included preliminary analysis and discussion in the revised manuscript. Page 24, line 504 to 513. Below is our response to the issues you raised and the corresponding modifications.

This study found that both cell morphology and mechanical properties undergo significant changes under fluid shear stress (FSS). The experimental results indicate that as the duration of FSS increases, HeLa cell height decreases, the morphology becomes more flattened, and the Young's modulus significantly decreases. This phenomenon is consistent with literature reports. For example, Novak et al. (as cited in reference [42] of our manuscript) observed that breast cancer cells exposed to shear stress in the tumor microenvironment (TME) exhibited significantly larger cell area, lower roundness, and a more motile phenotype. These stimulated cells were more proliferative than static controls and showed higher resistance to the anti-cancer drug paclitaxel. These results suggest that pulsatile shear stress promotes proliferation, invasive potential, chemotherapy resistance, and PLAU signaling in breast cancer cells. Fan et al. (as cited in reference [43] of our manuscript) observed through micropatterned cell culture that cells subjected to mechanical stimulation exhibited reduced height, a more flattened morphology, and cytoskeletal reorganization leading to a decrease in Young's modulus, making the cells softer. These changes in mechanical properties further influence cell functions such as migration, invasion, and nuclear gene expression.

To more deeply quantify the relationship between cell morphology and mechanical properties, we plan to systematically analyze multiple groups of cells under the same experimental conditions in future studies. This will provide more direct experimental evidence to reveal the interaction between cell morphology and mechanical properties.

Comment 7. The authors discuss potential mechanisms underlying the observed changes in cell mechanics, such as cytoskeletal reorganization, alterations in cell membrane fluidity, and changes in intracellular signal transduction. However, these discussions are rather speculative. Are there any experimental data or references to support these proposed mechanisms?

Response: We thank you for your valuable comments on the potential mechanisms underlying the changes in cellular mechanical properties. In the revised manuscript, we have added relevant references and discussions to enhance the credibility of these mechanisms. Page 3, line 58 to 71. Page 23, line 482 to 503, page 24, line514 to 518.

The following references and research findings support the potential mechanisms of changes in cellular mechanical properties:

1. Luo et al. (as cited in reference [40] of our manuscript): The study demonstrated that the stiffness of cancer cells and its changes during metastasis are crucial for understanding the pathophysiology of cancer cells and the mechanisms of cancer metastasis. The results showed that the flow environment affects the cytoskeleton, and the rearrangement of microfilaments significantly influences cellular mechanical properties.
2. Hayashi et al. (as cited in reference [39] of our manuscript): The study found that cancer cells exposed to normal and shear stresses undergo changes in morphology, structure, and mechanical properties. The lower stiffness of cancer cells facilitates their motility and metastatic potential during the transition from primary sites to metastatic sites.
3. Zhang et al. (as cited in reference [6] of our manuscript): An in vitro model was established to simulate the effects of blood shear stress on the adhesion of circulating tumor cells (CTCs) to brain endothelial cells. The results indicated that blood shear stress selectively enriches CTCs capable of stably adhering to brain endothelium, exhibiting stronger brain metastasis potential.
4. Oliveira et al. (as cited in reference [7] of our manuscript): Using open microfluidic chip technology, the study investigated the growth and differentiation of myoblasts (C2C12) under flow conditions, demonstrating the influence of the flow environment on cell differentiation.
5. Quesada et al. (as cited in reference [44] of our manuscript): The study found that under 10 dyn/cm² FSS, the expression of the proliferation marker Ki67 in MCF-7 cells was enhanced at the single-cell level, indicating that FSS increases protein phosphorylation associated with a more invasive phenotype. These findings help explain why cancer cells at metastatic sites are often more aggressive than primary breast cancer cells.

Supported by experimental data and literature, we have confirmed the important roles of cytoskeletal reorganization, changes in cell membrane fluidity, and intracellular signaling transduction in the fluid shear stress-induced changes in cellular mechanical properties. These mechanisms collectively regulate cellular mechanical responses, thereby influencing cell behavior and function. We believe these additions and improvements will make our study more scientifically rigorous. We sincerely appreciate your valuable suggestions.

Comment 8. the connection between the mechanical properties measured in vitro and the actual metastatic behavior of HeLa cells in vivo remains unclear. Could the authors discuss how these in vitro findings might translate to the in vivo context? Are there any in vivo studies that support the relationship between mechanical properties and metastasis?

Response: We thank you for raising the question regarding the connection between the mechanical properties measured in vitro and the actual metastatic behavior of HeLa cells in vivo. The discrepancy between in vitro and in vivo studies, as you pointed out, is indeed a significant scientific issue. We have included a detailed discussion of this aspect in the revised manuscript. Page4,line 72 to 82, Page24,line 485 to 503.

In this study, by measuring the mechanical properties of HeLa cells cultured in vitro, we found a significant correlation between the duration of FSS and the cells' Young's modulus. As the duration of FSS increased, the Young's modulus of the cells significantly decreased. This result indicates that fluid shear stress has a notable impact on the mechanical properties of HeLa cells. Research has shown that changes in the Young's modulus of cells are closely related to the increased metastatic potential of cancer cells. Lower cell stiffness is generally associated with more aggressive cancer cell behavior, likely due to the reorganization of the cytoskeleton under fluid shear stress [as cited in reference [40] of our manuscript]. Cancer cells with higher metastatic potential tend to have a lower Young's modulus. Reduced cell stiffness may facilitate the ability of cancer cells to penetrate blood vessel walls or the extracellular matrix (ECM), thereby promoting distant metastasis[as cited in reference [41] of our manuscript].

Although there are significant differences between the in vitro and in vivo environments, including the complexity of mechanical forces and the microenvironment, which may affect the translation of in vitro findings to in vivo conditions, some in vivo studies also support the association between the mechanical properties of cancer cells and their metastatic behavior. For instance, Lekka et al. (as cited in reference [as cited in reference [9] of our manuscript] of our manuscript) demonstrated that the Young's modulus of in vivo tumor tissue is significantly lower than that of normal tissue, consistent with the increased softness of cancer cells observed in vitro. This softness may facilitate cancer cells' ability to pass through tissue barriers, promoting invasion and metastasis. Cross et al. (as cited in reference [as cited in reference [10] of our manuscript] of our manuscript) measured the mechanical properties of cancer cells in the pleural fluid of cancer patients and found that the Young's modulus of metastatic cancer cells was significantly lower than that of normal cells. This clinical correlation suggests that the mechanical properties of cancer cells may play an important role in the metastatic process in vivo.

Although this study provides significant insights into the mechanical properties of cancer cells, certain limitations remain. In vitro experiments are typically conducted under static or single-variable conditions, making it difficult to fully replicate the complex TME in vivo, as well as the dynamically changing stiffness and composition of the ECM, both of which interact with cancer cells and influence tumor behavior. Therefore, future research needs to develop experimental models that more closely resemble the in vivo environment to better simulate the dynamic characteristics of the tumor microenvironment. Integrating in vitro findings with clinical data will enable a more comprehensive understanding of the role of cancer cell mechanical properties in cancer metastasis and provide a foundation for developing new therapeutic strategies.

Comment 9. Recent work in the field of Mechanical Properties of Cells should be cited in introduction, such as: https://doi.org/10.1016/j.biotechadv.2020.107648; DOI: 10.1017/S1431927622000290; DOI: 10.1039/D0LC00477D; doi: 10.3791/50497.

Response: It is a very good suggestion. This reference is very helpful for us to understand the measurement methods and applications of single-cell mechanical properties. We thank the reviewer for providing the relevant reference. We have added this reference in the revised manuscript, which is cited as [16].

**Reviewer 2:** The mansucript presents an experimental study on how cells change their mechanics after being exposed to hydrodynamic flow. This is an interesting and relevant subject, and the results are novel. That said, the study does suffer from some major issues that need to be improved, before publication can be recommendend. These are, in order of relevance:

Comment 1. It is not clearly said, but the way I understand the manuscript, each experiment is carried out for a single cell only. The error bars appear to stem from different measurement points on the same cell. Measuring AFM at different points on the same cell is highly important and a good point of the paper.

However, we can expect significant cell-to-cell variation as well. Therefore, it is highly important to make statistics on, at least, 5-10 different cells for each experimental situation.

Response: We thank the reviewer for their question. During the experiments, we actually measured multiple cells under each experimental condition. However, this was not explicitly stated in the experimental methods section of the original manuscript. To obtain statistically significant results, we performed morphological imaging on one group of cells and measured force curves for five different groups of cells under each experimental condition (the experimental methods section has been updated in the manuscript. Page 19, line 397 to 402，Page19，line 406-411). The Young's modulus measurement results for multiple groups of cells, obtained using AFM, are shown in Fig. 1.

**Fig. 1 Measurement of Young's modulus for multiple groups of cells using AFM**

We measured force curves for five groups of cells under each experimental condition and fitted 20 force curves from each group to determine the Young’s modulus of the cells. （Page22,line457-459.）The experimental results indicate that under identical fluid shear stress conditions, the Young’s modulus values of the cells tend to decrease with increased duration of exposure. As shown in Fig.2.

**Fig. 2 Measuring the Young's modulus of Hela cells subjected to different durations of fluid shear stress by Atomic Force Microscopy (AFM)**

Comment 2. I am missing a quantitative connection to earlier works. The HeLa cells used in the manuscript are a widely-used cell line and I am sure there exist earlier AFM (and possibly other) measurements of their elastic properties in the absence of flow. The authors should look for such data and compare their control experiment without flow to these works.

Response: Thank you very much for your thorough review of our research and your valuable feedback.

The issue you raised regarding establishing quantitative connections with earlier work is indeed crucial. In the revised manuscript, we have added discussions in the introduction sections to highlight the connections between our findings and prior studies. This includes key discoveries from research on the effects of FSS on cells, changes in cell morphology and mechanical properties, and potential mechanisms underlying these changes in cellular mechanical characteristics. Page 3, line 58 to 104.

We understand the importance of comparing the measurement data of cell elastic properties in the absence of flow with the work presented in this paper. However, due to differences in experimental conditions, measurement methods, and environmental parameters, direct comparisons may not yield clear conclusions. For example, Zhuang (as shown in Fig. 4 of the reference "Effect of substrate stiffness on the mechanical properties of cervical cancer cells", <https://doi.org/10.1016/j.abb.2022.109281>) investigated the effect of substrate stiffness on the mechanical properties of cervical cancer cells and found that an increase in substrate stiffness significantly enhanced the Young's modulus of SiHa cells. Differences in cell mechanical measurement methods also affect the results. When using atomic force microscopy (AFM) to measure cell mechanical properties, the AFM measurement model (such as contact mode, tapping mode, and PFQNM model) and the type of probe selected can lead to significant differences in the results. For example, Lü (as shown in Fig. 8 of the reference "Combining Atomic Force Microscopy With Optical Image Recognition for Rapid Measurements of Single-cell Mechanical Properties", [10.16476/j.pibb.2022.0456](http://dx.doi.org/10.16476/j.pibb.2022.0456)) explored the mechanical properties of living cells using AFM and found that under the same conditions, the Young's modulus of cells measured with a spherical tip probe was significantly lower than that measured with a conical tip probe.

The focus of this study is on the impact of a single variable (shear stress exposure time) on the mechanical properties of HeLa cells, aiming to reveal its changing trend. Therefore, direct comparison with existing data may lack quantitative correlation due to the interference of multiple variables. In the future, we plan to systematically compare different research findings on the basis of strictly controlled experimental conditions to further verify and expand the findings of this study.

Comment 3. The shear stress calculation in 2.3 1) seems to be an approximation. The exact flow field for a rectangular geometry is well-known, but is a somewhat lengthy expression containing an infinite convergent sum as given in standard texts on hydrodynamics. Please state which approximations were made to arrive at the simple form given in 2.3 1).

Response: Thank you for your valuable comments on the calculation of shear stress in the manuscript. We have provided a detailed explanation of the approximation involved in applying the relevant formulas in the revised manuscript. Page 10, line 196 to 201.

We used the classical fluid shear stress theoretical formula to calculate the wall shear stress in the parallel plate flow chamber and compared these results with numerical calculations. This formula assumes that the fluid is Newtonian and the flow is steady. In the actual flow chamber, due to the finite width and height of the channel, there is a certain velocity gradient variation at the boundaries of the channel. However, when b/a > 20, these boundary effects become negligible, and more than 85% of the surface is exposed to a uniform wall shear stress.

Moreover, the formula for calculating fluid shear stress employed in this study has been widely utilized in the computation of fluid shear stress in parallel-plate configurations. For instance, Ding et al. (reference: "Hemodynamic Shear Stress via ROS Modulates PCSK9 Expression in Human Vascular Endothelial and Smooth Muscle Cells and Along the Mouse Aorta," , doi: [10.1089/ars.2014.6054](https://doi.org/10.1089/ars.2014.6054) . page 2) applied this formula to calculate fluid shear stress, utilizing a parallel-plate flow chamber to investigate how fluid shear stress modulates the expression of PCSK9 in human vascular endothelial and smooth muscle cells through reactive oxygen species, as well as its distribution along the mouse aorta. Similarly, Wittkowske et al. (reference: "In Vitro Bone Cell Models: Impact of Fluid Shear Stress on Bone Formation," , <https://doi.org/10.3389/fbioe.2016.00087> , page 10) employed this formula to compute fluid shear stress, studying the effects of fluid shear stress on bone cell signaling, collagen deposition, and matrix mineralization in in vitro models.

These approximations enable us to estimate wall shear stress using a relatively simple formula. Furthermore, we have validated the reliability of the formula through numerical simulations. By comparing theoretical and numerical results, we have demonstrated the rationality of the formula's selection.

We tried our best to improve the manuscript and we appreciate for Editors/Reviewers’ warm work earnestly, and hope that the correction will meet with approval.

Once again, thank you very much for your comments and suggestions.

Yours sincerely

Yaoxian Wang

Department of Gynecological Radiotherapy, Harbin Medical University Cancer Hospital, No 150 Haping Road, Nangang District, Harbin 150081, Heilongjiang Province, China. Phone: +8618945000012.E-mail: [wyxxs012@126.com](mailto:wyxxs012@126.com).

On behalf of the co-authors
